# Modular Microphysiological System for Modeling of Biologic Barrier Function

**DOI:** 10.3389/fbioe.2020.581163

**Published:** 2020-11-12

**Authors:** Matthew Ishahak, Jordan Hill, Quratulain Amin, Laura Wubker, Adiel Hernandez, Alla Mitrofanova, Alexis Sloan, Alessia Fornoni, Ashutosh Agarwal

**Affiliations:** ^1^Department of Biomedical Engineering, University of Miami, Coral Gables, FL, United States; ^2^Department of Biochemistry & Molecular Biology, DJTMF Biomedical Nanotechnology Institute, University of Miami Miller School of Medicine, Miami, FL, United States; ^3^Katz Family Division of Nephrology and Hypertension, Department of Medicine, Peggy and Harold Katz Family Drug Discovery Center, University of Miami Miller School of Medicine, Miami, FL, United States

**Keywords:** organ-on-chip, lung-on-chip, glomerulus-on-chip, microphysiological system, microfluidic

## Abstract

Microphysiological systems, also known as organs-on-chips, are microfluidic devices designed to model human physiology *in vitro*. Polydimethylsiloxane (PDMS) is the most widely used material for organs-on-chips due to established microfabrication methods, and properties that make it suitable for biological applications such as low cytotoxicity, optical transparency, gas permeability. However, absorption of small molecules and leaching of uncrosslinked oligomers might hinder the adoption of PDMS-based organs-on-chips for drug discovery assays. Here, we have engineered a modular, PDMS-free microphysiological system that is capable of recapitulating biologic barrier functions commonly demonstrated in PDMS-based devices. Our microphysiological system is comprised of a microfluidic chip to house cell cultures and pneumatic microfluidic pumps to drive flow with programmable pressure and shear stress. The modular architecture and programmable pumps enabled us to model multiple *in vivo* microenvironments. First, we demonstrate the ability to generate cyclic strain on the culture membrane and establish a model of the alveolar air-liquid interface. Next, we utilized three-dimensional finite element analysis modeling to characterize the fluid dynamics within the device and develop a model of the pressure-driven filtration that occurs at the glomerular filtration barrier. Finally, we demonstrate that our model can be used to recapitulate sphingolipid induced kidney injury. Together, our results demonstrate that a multifunctional and modular microphysiological system can be deployed without the use of PDMS. Further, the bio-inert plastic used in our microfluidic device is amenable to various established, high-throughput manufacturing techniques, such as injection molding. As a result, the development plastic organs-on-chips provides an avenue to meet the increasing demand for organ-on-chip technology.

## Introduction

Microphysiological systems (MPS), commonly referred to as organs-on-chips or tissue chips, have emerged as a novel approach to create *in vitro* models of normal and disease physiology. By incorporating human cells into microfluidic devices that recapitulate physiological tissue architecture and dynamic *in vivo* stimuli, MPS have promised to address the high attrition rate of compounds in drug development by providing a more human-relevant tool to identify therapeutic targets and assess drug toxicity (Bhatia and Ingber, 2014). The rapid development of MPS has been greatly influenced by the widespread use of polydimethylsiloxane (PDMS) as a biocompatible material for microfluidic devices (Duffy et al., 1998; McDonald and Whitesides, 2002; Huh et al., 2013). PDMS demonstrated a number of advantages for early biological applications, including low cytotoxicity, optical transparency, gas permeability, and ease of microfabrication (Sia and Whitesides, 2003). One of the earliest MPS, a lung-on-a-chip, validated PDMS for applications that involve recreating the dynamic flow and mechanical stretching of the *in vivo* alveolar air-liquid interface (ALI) (Huh et al., 2007, 2010). However, the widespread adoption of MPS has been hampered by some of the innate properties of PDMS (Berthier et al., 2012). Of these drawbacks, the absorption of small molecules and leaching of uncrosslinked oligomers have provided the greatest hindrance to the adoption of PDMS-based MPS in the context of drug discover assays (van Meer et al., 2017).

Due to the long history of microfluidic technology, there is extensive literature on the fabrication and use of plastic microfluidic devices, which may serve as an alternative to PDMS for microphysiological systems (Becker and Locascio, 2002; Guckenberger et al., 2015). Poly(methyl methacrylate) (PMMA), a transparent thermoplastic, is not only amenable to a number of microfluidic fabrication techniques, but also highly biocompatible and less absorptive than PDMS. Previously, we have demonstrated the feasibility of a PMMA fluidic platform for biologic applications through the functional assessment and optogenetic control of pancreatic islets (Lenguito et al., 2017). However, designing fluidic channels to recapitulate *in vivo* barriers, such as the alveolar air liquid interface ALI or glomerular filtration barrier (GFB), while maintaining a resealable format is difficult due to the limitations of the subtractive rapid prototyping (SRP) technique employed to fabricate PMMA-based MPS.

Here, we have overcome this limitation and engineered a modular MPS using PMMA to recapitulate the *in vivo* microenvironment of biologic barriers. The two-part microfluidic chip is comprised apical and basal channels separated by a removable porous membrane. We demonstrate that static co-culture models of the lung ALI and GFB can be transferred to the MPS and exposed to physiomimetic dynamic stimuli. This approach aims to simplify cell culture and seeding methods within the microfluidic device and minimize the time to achieve functional constructs within the MPS. Together, these results establish that a robust MPS platform can be developed from commonly used plastics that provide an avenue to meet the increasing demand for organ-on-chip technology.

## Materials and Methods

### Design and Assembly of PDMS-Free Microfluidic Chip

Apical and basal flow channels were incorporated into a two-part, resealable form-factor, based on our previously published platform for interrogation of pancreatic islets. Computer-aided design (CAD) software (SolidWorks, Dassault Systèmes) was used to generate three-dimensional parts and assemblies of the fluidic chip design. SRP of CAD designs was used to fabricate fluidic chips. Briefly, device features were milled from optically-clear, UV-resistant acrylic (0.125° thick, McMaster-Carr) using a computer numerical control milling machine (MODELA MDX-540, Roland). A 30W CO2 laser machine (Legend Helix, EpilogLaser) was used to laser cut the final form-factor of the chip from the milled workpieces. A fluoropolymer membrane was bonded to the underside of the bottom piece using a silicone adhesive. Custom gaskets were fabricated using a two-part silicone epoxy (Duraseal 1533, Cotronics Corp.). To improve the ease and speed of assembly, the gaskets were bonded to the top piece of the fluidic chip using a 100 μm thick differentially-coated polyester adhesive film (PS-1340, Polymer Science). The fully fabricated fluidic chip is then clamped in a chip holder (Fluidic Connect PRO, Micronit) to create a fluidic seal and introduce tubing connections.

### Flow Profile Characterization

The fully fabricated fluidic chip is clamped in a chip holder (Fluidic Connect PRO, Micronit) to create a reversible fluidic seal and introduce tubing connections. Two microfluidic pressure pumps (Flow-EZ, Fluigent) were used to independently provide flow to the apical and basal flow channels. Flow rate was continuously measured at the outlet of the fluidic chip using liquid flow meter microsensor (LG16, Sensirion). The average flow rate was calculated from 60s recordings at pressures ranging from 0 to 150 mbar.

From flow rate measurements, the fluid shear stress τ()n the apical and basal sides of the membrane was calculated using the following equation:

$$\tau =\frac{6\,\mu \,\text{Q}}{{\text{bh}}^{2}}$$

ere μ is the fluid viscosity, *Q* is the volumetric flow rate, *b* is the channel width at the center of the culture well, and *h* is the height of the culture well (0.5 mm). In the present design, the width of the culture well for the apical and basal flow channels was 6 and 4.5 mm, respectively, and the height of the channels.

### Computational Fluid Dynamics

Computational modeling of fluid dynamics was performed using the FEM software, COMSOL Multiphysics 5.0. Three-dimensional models of the fluidic channels were imported into COMSOL from SolidWorks as a Parasolid file. The Free and Porous Media Flow physics module was used to solve for velocity and pressure fields of single-phase flow in the channels and the porous membrane separating the channels, simultaneously. Fluid flow was modeled as the incompressible flow of water (density = 1000 kg/m^3^ and dynamic viscosity = 0.001 Pa⋅s) governed by the Navier-Stokes and Brinkman equations. The permeability parameter of the membrane was approximated based on the hydraulic-electrical circuit analogy (Sip et al., 2014; Chung et al., 2018). This approach is based on the similarities of Hagen–Poiseuille’s law for fluid flow and Ohm’s law for electrical current flow. Assuming each pore of the membrane is a circular pipe, the fluidic resistance of a single pore (*R*_*pore*_) can be calculated:

$${\text{R}}_{\mathrm{pore}}=\frac{8\,\mu \,\text{L}}{\pi \,{\text{R}}^{4}}$$

Where μ is the viscosity of the fluid, L is the thickness of the membrane, and R is the radius of the pore. The fluidic resistance of the whole membrane (*R*_*memb*_) can then be calculated from the area (*A*) and porosity (ρ*_*pore*_*) of the membrane:

$${\text{R}}_{\mathrm{memb}}=\frac{{\text{R}}_{\mathrm{pore}}}{\text{A}\,\rho_{\mathrm{pore}}}$$

Using Ohm’s law and the above equations, the permeability parameter (*k*) can then be expressed as a function of the porosity and pore radius:

$$\text{k}=\frac{\pi \,\rho \,{\text{R}}^{4}}{8}$$

A stationary solver was implemented to determine the steady state solution of the velocity and pressure fields. Flow rates were derived from the 3D computational models by taking the surface integral of the velocity field across specific regions.

### Characterization of Membrane Strain

Bi-axial strain applied to the membrane was calculated based on the membrane deflection due to applied pressure. Membrane deflection was measured using the perfect focus system on a TI-Eclipse microscope (Nikon). First, the pump for basal flow channel was set to 80 mbar to perfuse phosphate buffered saline through the basal channel. Next, the initial focal distance of the center of the membrane was recorded. Then, the pressure for the apical flow channel pump was increased from 0 to 315 mbar in 15 mbar increments. The focal distance at each applied pressure was recorded and membrane deflection was calculated as the change from the initial focal distance. The surface of the area of the membrane could then be calculated by assuming a semi-ellipsoid geometry:

$$SA=2\;\text{π}*\;{\left[\frac{{\left(a\;*\;b\right)}^{1.6075}+{\left(a\;*\;c\right)}^{1.6075}+{\left(b\;*\;c\right)}^{1.6075}}{3}\right]}^{\frac{1}{1.6075}}+(\text{π}\;*\;b\;*\;c)$$

Were *a* is the membrane deflection and *b* and *c* are the major and minor semi-axes, respectively. Based on the change of surface area, the bi-axial surface expansion (ε_SA_) can be calculated (Guenat and Berthiaume, 2018):

$$\varepsilon_{\mathrm{SA}}=\frac{{\text{SA}}_{f}-{\text{SA}}_{0}}{{\text{SA}}_{0}}$$

Were *SA*_*0*_s the initial surface area of the membrane when no pressure is applied and *SA*_f_s the surface area of the membrane calculated based on the membrane deflection.

### TEER Measurement

Integrity of the cellular layers was assessed by transendothelial/transepithelial electrical resistance (TEER) during static culture on transwells. Resistance measurements were obtained using an epithelial volt/ohm meter (EVOM2, World Precision Instruments) equipped with handheld chopstick electrodes (STX2, World Precision Instruments). To avoid variability in measurements, test samples were brought to room temperature and electrodes were held in place using a universal probe stand. TEER values were obtained using the Ohm’s Law Method (Srinivasan et al., 2015). Briefly, the resistance of the cellular layer (*R*_cells_) first calculated:

$${\text{R}}_{\mathrm{cells}}={\text{R}}_{\mathrm{meas}}-{\text{R}}_{\mathrm{blank}}$$

Were *R*_meas_ is the resistance measurement of the sample, and *R*_blank_ is the resistance measurement of a transwell membrane without cells. Then the TEER value was calculated by:

$$\text{TEER}={\text{R}}_{\mathrm{cells}}\times {\text{M}}_{\mathrm{area}}$$

Were *M*_area_ is 0.336 cm^2^, the area of the transwell membrane.

### Culture of Alveolar-Capillary Interface

Human alveolar epithelial cells (AECs; ATCC) were propagated according to the manufacturer’s instructions in ATCC Modified RPMI1640 (Thermo Fisher). Human lung microvascular endothelial cells (LMECs, Lonza) were cultured in EGM2 with the manufacturer’s supplements.

Recapitulation of the alveolar-capillary interface was achieved by generating an air-liquid interface (ALI) co-culture of AECs and LMECs using methods previously described, with minor modifications (Huh et al., 2010, 2013). Briefly, transwell inserts with 0.4 μm pores (Greiner Bio-One), were coated with fibronectin (Sigma), diluted to 5 μg/mL. LMECs and AECs were seeded on inserts as described for GFB co-culture. On day 3 of the co-culture, the media for the AECs was supplemented with 1 μM dexamethasone. Once cells were confluent, after about 7 days, ALI was induced and the media in the lower compartment was changed to a 50/50 mix of EGM2 and ATCC Modified RPMI supplemented with 1 μM dexamethasone.

### Glomerular Filtration Barrier Co-culture

Conditionally immortalized human podocytes (CiPodos) were cultured as previously described, with minor modifications (Saleem et al., 2002). Briefly, CiPodos were propagated on collagen-coated flasks in permissive conditions (33°C, 5% CO_2_) in RPMI1640 media supplemented with penicillin, streptomycin, insulin, transferrin, selenium, and 10% fetal bovine serum.

Primary human glomerular microvascular endothelial cells (GMECs; Cell Systems) were culture per the manufacturer in complete endothelial cell growth media 2 (PromoCell). Briefly, GMECs were propagated on T75 flasks coated with Attachment Factor (Cell Systems) with media replaced every other day.

Recapitulation of the glomerular filtration barrier (GFB) was achieved by co-culturing CiPodos and GMECs on opposite sides of transwell insert with 3 μm pores (Greiner Bio-One). First, inserts were coated with collagen type I from rat tail (Corning) diluted to 0.1 mg/mL. Then, inserts were inverted and GMECs were seeded on the underside of the insert at a density of 50,000 cells per insert. The inverted inserts were then incubated for approximately 2 h to allow for GMECs to adhere. Finally, the inserts were placed in a 24-well culture plate and CiPodos were seeded in the upper compartment at a density of 50,000 cells per insert. Cells were fed with their respective media every other day.

### Immunocytochemistry

Immunoctyochemistry was performed by placing membranes removed from the MPS into a series of 1.5 mL Eppendorf tubes. Cells were first fixed in 4% paraformaldehyde and permeabilized in 0.3% Triton-X. Cells were incubated with primary antibodies, diluted 1:400, for 1 h followed by 3 sequential washes in PBS. Cells were incubated with secondary antibodies, diluted 1:500, for 1 h. Nuclei were stained with either ProLong Diamond Antifade Mountant with DAPI (Invtriogen) or Hoescht (1:2000; Life Technologies). Images were acquired on either a Ti Eclipse widefield microscope (Nikon) or a Zeiss LSM confocal microscope. Image analysis was performed using ImageJ Fiji (Schindelin et al., 2012).

### Filtration Assay

For the filtration assay, membranes were cut from their supports after 7 days of culture at 37°C and bonded into a microfluidic device. Two microfluidic pressure pumps (Fluigent, Flow-EZ) were used to drive flow and generate a physiomimetic pressure gradient for 1 h. A pressure of 80 mbar (approximately 60 mmHg) was applied to the basal flow channel and 20 mbar (approximately 15 mmHg) was applied to apical flow channel to perfuse PBS through the system. The basal flow channel was supplemented with 100 μg/mL FITC-conjugated inulin (Sigma-Aldrich, F3272) and/or 2 mg/mL bovine serum albumin (Sigma-Aldrich, A2153). The concentration of albumin was measured in the outflow from each channel based on the absorbance at 280 nm using a NanoDrop UV-Vis Spectrophotometer (Thermo Fisher Scientific). The fluorescence intensity of inulin in the outflow from each channel was measured using a multimode microplate reader (Beckman-Coulter, DTX 880). The amount of albumin or inulin filtered from the basal channel to the apical channel was calculated using the equation for renal clearance (Musah et al., 2017):

Filtration=([A]×AV)/[B]

Were [*A*] = concentration in apical outlet, *AV* = apical volume collected, and [*B*] = concentration in basal outlet. Filtration of albumin and inulin was normalized to the filtration observed on a blank membrane without cells according to the following equation:

$$\mathrm{Normalized}\,\text{Filtration}=\frac{{\text{Filtration}}_{\mathrm{cells}}}{{\text{Filtration}}_{\mathrm{blank}}}$$

### Sphingolipid Exposure

The ability of podocyte cultures to filter albumin and inulin was assessed after a 1-hour exposure to sphingosine-1-phosphate (S1P), which has previously been shown to cause proteinuria (Schümann et al., 2015). As a control, podocyte cultures not exposed to S1P were also assessed for filtration function. To expose podocytes to S1P, culture media was replaced with RMPI media supplemented with 5 μM S1P 1 h prior to cutting membranes from the supports for bonding into the MPS for filtration assay.

## Results

### Development of Modular, PDMS-Free MPS

**FIGURE 1:**
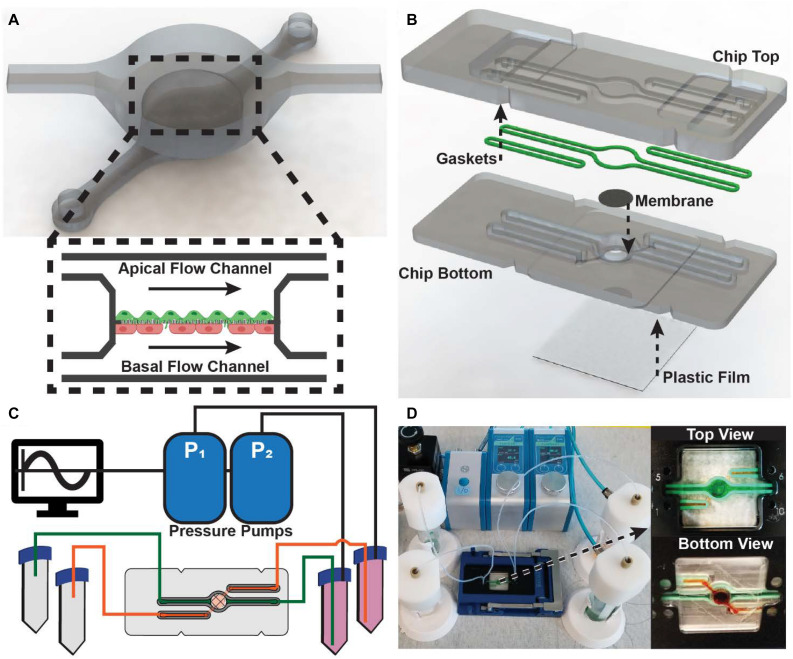
Design and assembly of the modular MPS: **(A)** 3D render of fluidic channel geometry implemented in microfluidic chip **(Inset)** Schematic representation of internal culture well with independent apical and basal channels and a co-culture of endothelial and epithelial cells supported by a porous membrane. **(B)** Exploded view of the microfluidic chip comprised of a top and bottom piece, gaskets, porous support membrane, and an oxygen-permeable plastic film. The arrows indicate how the components are bonded into the chip. **(C)** Schematic representation of the MPS comprised of two pressure pumps controlled by a computer, media reservoirs, and the microfluidic chip. **(D)** Fully assembled and running MPS. (Insets) Top and bottom view showing leak-free perfusion of dyed water through the microfluidic chip.

The design of the fluidic chip used in our modular platform, which is analogous to many PDMS-based organs-on-chips, was developed to house an epithelial/endothelial cellular co-culture (Figure 1A). Using a three-dimensional (3D) computer-aided design (CAD), a model of the chip was developed to scale to ensure the top and bottom pieces of the chip were compatible and that a porous membrane support would fit in to the culture well (Figure 1B). Unlike additive fabrication methods, such as 3D printing and soft lithography, the SRP approach makes fabrication of closed channels in the z-direction difficult. With PDMS-based organs-on-chips, irreversibly bonding two pieces of PDMS is a common solution to create closed fluidic channels. However, this reduces the modularity of the device. SRP, on the other hand allows for fast, low cost fabrication of microfluidic devices from commonly used plastics that can be are resealable. To maintain resealable form-factor of the chip design, the basal flow channel was closed by adhering a fluoropolymer membrane to the underside of the bottom piece. This membrane not only provides a thin, clear window for optical access, but also enables gas permeability in the culture well of the chip.

To recapitulate the dynamic stimuli that occur *in vivo*, the organ-on-chip platform were modular pneumatic microfluidic pumps implemented to drive flow. Using the microfluidic automation tool software, the pumps can generate physiomimetic waveforms that drive independent perfusion of the apical and basal flow channels (Figure 1C). To validate the design of the modular organ-on-chip platform, the fluidic chip was tested for leaks. Using a commercially available chip holder, the fluidic chip could be sealed and easily connected to the media reservoirs. Both the apical and basal flow channels were observed to operate without leaks (Figure 1D).

### Recapitulating the Alveolar ALI Microenvironment

**FIGURE 2:**
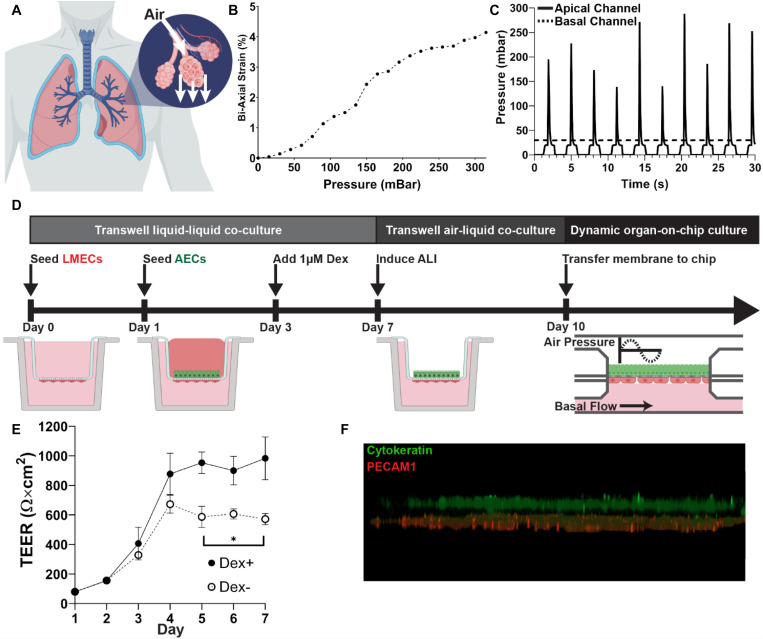
Recapitulating alveolar microenvironment: **(A)** Cartoon of alveolar expansion during inspiration. Created with BioRender.com. **(B)** Calculated bi-axial strain in response to applied pressure in the microfluidic chip. **(C)** Application of a cyclic pressure in the apical channel using a sine wave to mimic breathing at approximately 20 breaths per minute. A constant pressure was applied to the basal channel to drive the flow of PBS. **(D)** Workflow for development of ALI co-culture. Endothelial cells (LMECs) were first seeded on the basal surface of a transwell culture insert, followed alveolar epithelial cells (AECs). Dexamethasone was added to the apical chamber on Day 3 to enhance the epithelial cell barrier. On Day 7, the ALI was induced by removing media from the apical chamber. On Day 10, the membrane was cut from the transwell support and bonded into the microfluidic chip and exposed to dynamic strain. **(E)** TEER measurements during liquid-liquid co-culture in the transwell with (Dex +) and without (Dex-) dexamethasone. Statistical analysis by unpaired *t*-test (*N* = 3). **(F)** 3D visualization of fluorescent *z*-stack after culture for 24 h under cyclic pressure exposure in the MPS. Cytokeratin (green) staining shows the alveolar endothelial cells on the apical side of the membrane, while PECAM1 (red) staining indicates the lung microvascular endothelial cells on the basal side.

To demonstrate the ability to model *in vivo* microenvironments, we first sought to recapitulate the ALI found at the alveolar-capillary barrier in the lungs. A key feature of the alveolar microenvironment is the cyclic stretching of the alveolar capsule during inspiration (Figure 2A). Therefore, we aimed to apply mechanical force to stretch the membrane through the apical flow channel of the MPS. By perfusing the basal flow channel with phosphate buffered saline (PBS) and applying air pressure through the apical pneumatic pump, deflection of the porous membrane was observed, and that the degree of deflection was proportional to the pressure applied. From the measured membrane deflection, bi-axial strain could be calculated by assuming a semi-ellipsoid shape of the deformed membrane (Figure 2B). The maximum bi-axial strain applied to the membrane without cells was just 4%, slightly lower than 4–12% linear strain thought to occur *in vivo* at the alveolar ALI (Guenat and Berthiaume, 2018). To model the cyclic breathing cycle, a sine wave with an amplitude of 345 mbar and a frequency of 0.33 Hz, corresponding to 20 breaths per minute, was generated in the apical flow channel while the basal flow channel pressure remained constant. The rapid switching of pressure resulted in a pressure profile resembling a human breathing profile more than a sine wave (Figure 2C).

Next, an ALI co-culture of lung endothelial and epithelial cells was incorporated in the MPS. Recapitulation of the alveolar-capillary interface was achieved by generating an air-liquid interface (ALI) co-culture of AECs and LMECs using methods previously described, with minor modifications (Figure 2D; Huh et al., 2010, 2013). TEER measurements were recorded during the transwell liquid-liquid co-culture (Day 0 to Day 7). Following the addition of dexamethasone on Day 3, a significantly higher TEER value was observed (Figure 2E). This is a result of increased mucus production, due to the role of dexamethasone in mucin production (Lu et al., 2005). On Day 10, ALI co-cultures were transferred into the MPS. Physiomimetic culture conditions were generated by the cyclic application of air pressure to the apical channel. Immunocytochemistry revealed that both endothelial cells, indicated by PECAM1, and epithelial cells, indicated by cytokeratin, were maintained within the MPS under physiomimetic culture conditions (Figure 2F).

### Characterization Flow Profile and Ultrafiltration

**FIGURE 3:**
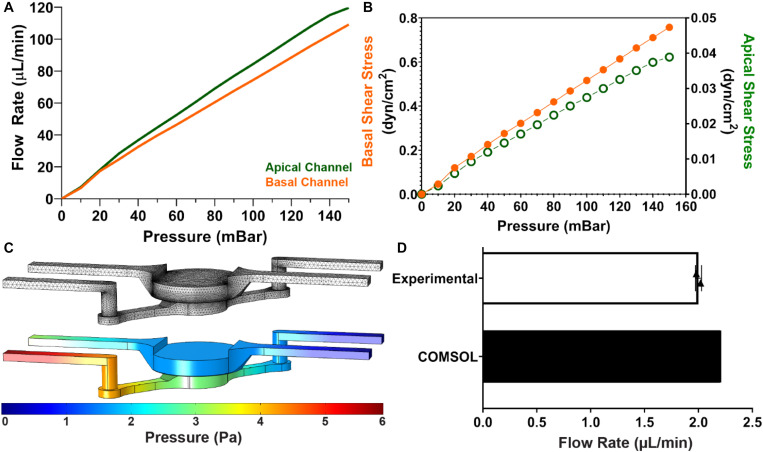
Characterization of Fluid Dynamics in MPS: **(A)** Cartoon of how glomerular anatomy was modeled within the MPS. Created with BioRender.com. **(B)** Calculated wall fluid shear stress at the center of the culture well in apical and basal channels. Here, the differences in geometry results in an order of magnitude difference in shear stress. **(C)** 3D computational fluid dynamics simulation in microfluidic chip. The top image is the mesh implemented for finite element analysis. The bottom image is the 3D volume view of steady state pressure field in MPS at 100 μL/min inlet flow rate in the apical and basal channels. **(D)** Fluid flow rate through the membrane separating the apical and basal flow channels determine experimentally and computationally using COMSOL.

An advantage the modular MPS design, is the ability to model multiple without significantly altering the device. As a result, we sought to also model a liquid-liquid barrier. First, we determined the fluid shear stress at the apical and basal boundaries of the membrane separating the two fluidic channels. This achieved by characterizing the flow rate as a function of the applied pressure. As expected, based on the electronic-hydraulic analogy, a linear relation between flow rate and applied pressure was observed (Figure 3A). This can be attributed to the constant fluidic resistance of the channels and the constant applied pressure. *In vivo*, vascular flow is often much faster than interstitial flow. This exposes endothelial cells to high shear stress flow, while epithelial cells experience much less shear stress. Due to the larger channel geometry, the flow rate in the apical channel was slightly higher than the flow rate in the basal channel. However, the fluid velocity and the resulting shear stress at the surface of the membrane, was about an order of magnitude larger in the basal channel than the wall shear stress calculated in the basal channel (Figure 3B). Therefore, the MPS design can model the fluid dynamics observed *in vivo.*

Based on these results, it was hypothesized that a pressure gradient would develop across the membrane between the two fluidic channels. A 3D computational model of the velocity and pressure fields within the microfluidic device at 100 μL/min, implemented in COMSOL, confirmed this (Figure 3C). The stationary solver calculated the velocity and pressure fields in 129 s using a normal physics-controlled mesh consisting of 262874 elements. The increased hydrostatic pressure in the basal channel suggests that ultrafiltration should occur within the device. By computing the surface integral of the velocity profile at the membrane surface, a flow rate of 2.21 μL/min was observed in the computational model. This result was confirmed experimentally by comparing the volume change in the apical and basal reservoirs after continuous perfusion overnight. In this setup, a peristaltic pump was implemented to recirculate fluid, ensure a constant flow rate, and mimic steady state conditions (Figure 3D).

### Functional Modeling of the Glomerular Filtration Barrier

**FIGURE 4:**
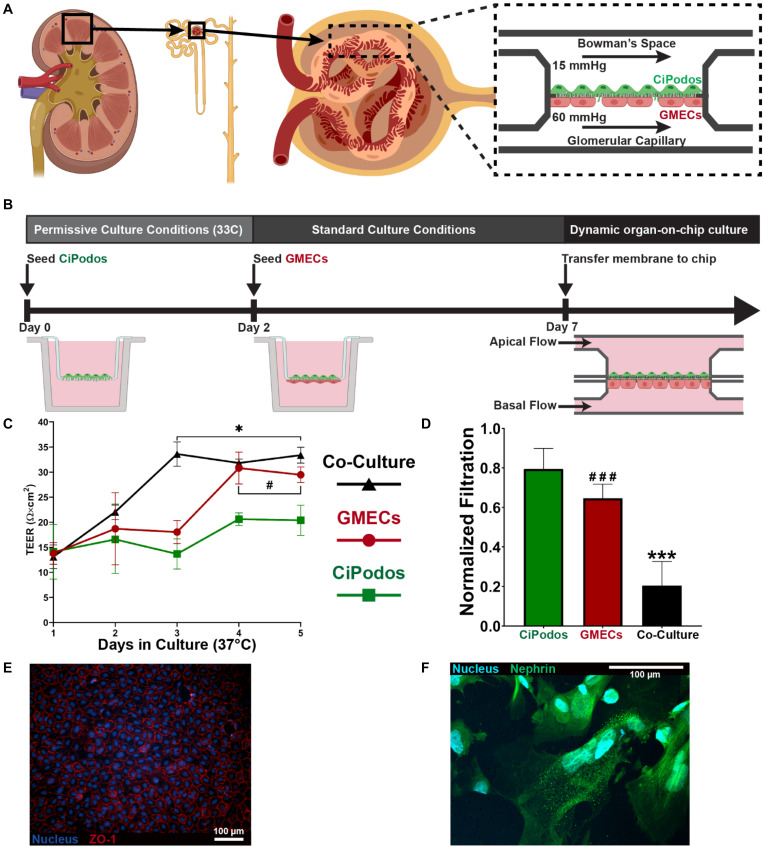
Modeling glomerular filtration barrier; **(A)** Cartoon of how glomerular anatomy was modeled within the MPS **(B)** Schematic representation of the workflow for the development of the co-culture model of the GFB. **(C)** TEER measurements of podocytes only (Green), endothelial cells only (Red), and co-culture (black) during static culture on a transwell insert over the course of 5 days. Data presented as mean ± standard deviation. Statistical analysis by unpaired t-test (*N* = 3; * indicates Co-Culture vs. CiPodos; # indicates GMECs vs. CiPodos). *p < 0.05, ^#^*p* < 0.05. **(D)** Urinary filtration of albumin in 1 h on the microfluidic device. The significant decrease in the urinary filtration of albumin between the co-culture, despite similar TEER values, indicates a functional barrier as opposed to a physical barrier. Statistical analysis by one-way ANOVA with Tukey’s multiple comparisons (*N* = 5; * indicates Co-Culture vs. CiPodos; # indicates GMECs vs. CiPodos). ***p < 0.0001, ^###^*p* < 0.0001. **(E)** Tight junctions observed in endothelial cells following culture on the MPS **(F)** Presence of nephrin in CiPodos after culture in the MPS.

To validate that the microfluidic device can recapitulate a functional biologic barrier, a model of the GFB was developed and assessed for albumin filtration. The filtration unit of the kidney, the nephron, begins in the highly selective glomerulus. The GFB is comprised of fenestrated glomerular endothelium and the glomerular epithelial cells, podocytes, separated by the unique glomerular basement membrane (Figure 4A). A co-culture of CiPodos and GMECs was established using a permeable membrane cell culture insert (Figure 4B). CiPodos were first seeded on the apical side of the membrane and cultured for 48 h under permissive conditions (33°C, 5% CO_2_). GMECs were then seeded on the basal side of the membrane and the co-culture were shifted to standard culture conditions (37°C, 5% CO_2_).

During the static culture in standard conditions, the integrity of the cellular layers was monitored through TEER measurements. TEER measures the electrical resistance across cellular layers to confirm barrier integrity of prior to evaluation of permeability to specific molecules (Srinivasan et al., 2015). Generally, TEER measurement provides a way to approximate cell coverage of the membrane. Under standard culture conditions, the CiPodos terminally differentiate and stop proliferating. As a result, only a slight increase in TEER is observed when podocytes are cultured alone (Figure 4C). On the other hand, GMECs will continue to proliferate when cultured at 37°C, resulting in a more confluent cellular barrier (Figure 4C). Therefore, a larger increase in TEER is observed over the course of the culture. As expected, a large increase in TEER was also observed in the co-culture of CiPodos and GMECs. Further, the final TEER measurement of the co-culture was just slightly higher than the TEER measurement of GMECs alone.

To assess the ability of the co-culture model to recapitulate the physiological function of the GFB, an albumin filtration assay was performed. Physiomimetic pressure driven flow was applied to perfusion PBS through the device for 1 h. Based on the results of the flow characterization, we were able to recreate the hydrostatic pressure of the glomerular microenvironment by applying 80 mbar (approximately 60 mmHg) to the basal flow channel and 20 mbar (approximately 15 mmHg) to apical flow channel. The basal flow channel was supplemented with bovine serum albumin (2 mg/mL) to mimic the oncotic pressure gradient. Compared to a blank membrane (i.e., no cells), all three cell models significantly reduced the urinary filtration of albumin (Figure 4D). Interestingly, the urinary filtration of albumin was also significantly less in the co-culture model compared to GMECs only, despite similar TEER measurements. These results highlight the importance of the cross-communication between endothelial cells and podocytes, which has been shown to be crucial in the regulation of the GFB (Dimke et al., 2015). Immunocytochemistry revealed that the functional proteins were present in the cells on both the basal and apical sides of the membrane. In the endothelial cells, ZO-1 staining indicated the tight junctions (Figure 4E). Nephrin, a protein required for proper GFB function, was observed in the CiPodos on the apical side of the membrane (Figure 4F).

### Sphingolipid Exposure Alters Albumin Filtration

**FIGURE 5:**
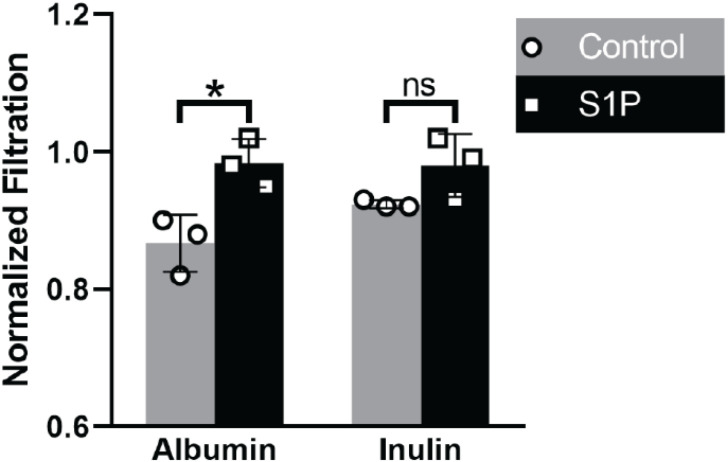
Effect of S1P on Podocyte Filtration: Urinary filtration of albumin was increased as a result of exposure to S1P. Urinary filtration of inulin, which is freely filtered at the GFB, was not significantly altered. Statistical analysis by unpaired *t*-test (*N* = 3, *p < 0.05).

Sphingolipids have been shown to be important second messengers in cellular processes such has growth and apoptosis (Olivera and Spiegel, 1993; Kaipia et al., 1996). S1P, which is generated by phosphorylation of sphingosine, has been indicated to play a role in many diseases, including diabetic kidney disease (Maceyka et al., 2012; Merscher and Fornoni, 2014). Podocytes play a significant role in the filtration of albumin at the GFB (Brinkkoetter et al., 2013). Dysfunction of podocytes can lead to albuminuria, which is a hallmark of kidney disease. In addition, deficiency of sphingosine 1 phosphate lyase in mice (Schümann et al., 2015) and humans (Lovric et al., 2017) has been associated to the development of podocyte injury and albuminuria. To elucidate the effect of S1P podocytes’ ability to maintain a functional filtration barrier, cells were exposure to 5 μM S1P for 1 h and then assessed for filtration function. A significant increase the urinary filtration of albumin was observed after just 1 h of exposure to 5 μM S1P (Figure 5). Urinary filtration of inulin, which is freely filtered at the GFB, was not significantly altered due to exposure to S1P. The observed change in albumin filtration after exposure to S1P suggests that exposure to this specific active sphingolipid may be sufficient to cause podocyte dysfunction, resulting in albuminuria.

## Discussion and Conclusion

For many organs, such as the lung and kidney, the functional unit is a biologic barrier. As a result, modeling of barrier function has been the primary focus of many organ-on-chip devices. The overall design of the microfluidic device is analogous to many biologic barriers and organ-on-chip devices. One the earliest organs-on-chips, a lung-on-chip, was developed using PDMS (Huh et al., 2010). However, PDMS absorption of small molecules is problematic for drug discovery assays (van Meer et al., 2017). Two independent flow channels are separated by a porous membrane that can support the culture of an endothelial and epithelial cell layer. The fabrication of stacked channels is relatively straightforward when using additive manufacturing techniques, such as soft lithography or 3D printing. Through these methods, devices can be assembled in layers either through bonding or material deposition. However, SRP, like all fabrication methods, offers benefits and challenges for the fabrication of microfluidic devices. Since material must be removed, it can be difficult to achieve closed channels at different heights within the device. This challenge has been overcome through the resealable form-factor implemented in the device design and the incorporation of an oxygen-permeable membrane to seal the basal channel. Although, a silicone-based adhesive is utilized in this design, it is not in direct contact with fluid which significantly reduces the potential for absorption or leaching. Further, SRP via micromilling presents advantages in terms of start-up cost, time, and versatility (Guckenberger et al., 2015). Specifically, the acrylic MPS described in this work can be completely fabricated relatively quickly (approximately 6 h) compared to similar PDMS microfluidic chips (approximately 40 h) (Huh et al., 2013). To achieve the same precision and optical using 3D printing would require more expensive and specialized equipment and materials. Here, we demonstrate that these advantages of SRP can be utilized to fabricate a highly functional MPS.

Independent perfusion of the two-channels was achieved using two microfluidic pressure pumps. Pressure-driven flow control provides many advantages over other methods, including stable, pulseless flow, fast response time, and precise flow rate control. Further, the use of pressurized air facilitated the implementation of cyclic air flow to stretch the porous membrane and mimic the human breathing cycle. We demonstrated the ability to create an air-liquid interface co-culture model that can be incorporated into the microfluidic device. Pressure-driven flow also facilitated the generation of hydrostatic pressure gradients, like those that are found at the glomerular filtration barrier. Together, the versatility of the platform enables recapitulation of multiple *in vivo* microenvironments.

The final proof-of-concept experiment for the microfluidic device design was to model the filtration function of the glomerulus. This was achieved by culturing the glomerular endothelial and epithelial cells on a porous membrane which could be transferred into the MPS. We also demonstrated that the ability of podocytes to effectively filter albumin can be directly modulated by exposure to sphingolipids. A significant limitation of traditional static culture systems is the inability to recapitulate organ-level function. The microfluidic device to model biologic barriers enables filtration assays to functionally assess podocytes. Despite the model being limited to just podocytes in the present study, a significant increase was observed in urinary filtration of albumin after exposure to S1P, thus setting the basis for the development of an assay to be utilized for clinically relevant drug discovery in proteinuric kidney diseases. Future studies will focus on recapitulating a more complete GFB by incorporating endothelial cells and a basement membrane. Albumin is one of the most abundant proteins in human blood and the loss of albumin via the urine is a key clinical marker for glomerular diseases. We demonstrate that under physiologically relevant pressures, the co-culture of GMECs and CiPodos can effectively prevent urinary filtration of albumin. In the present model, however, urinary filtration of albumin was not minimized to levels that would be translatable to a clinical setting. This may be attributed to the cell source or the lack of a basement membrane components. The GFB is a complex, highly selective filter and future work will be necessary to improve upon the biologic components.

In conclusion, we report the design and fabrication of a PDMS-free microfluidic device that can recapitulate many of the dynamic stimuli originally described for organs-on-chips. We implemented a resealable form-factor to incorporate a porous1membrane that can support the culture of an endothelial and epithelial cell layer and an oxygen-permeable membrane to seal the basal channel. The versatility of the design was demonstrated by constructing an alveolar air-liquid interface with cyclic stretching to mimic lung breathing and by modeling selective filtration by the kidney at the GFB with a physiomimetic pressure gradient. We establish the utility of the modular MPS design by recapitulating sphingolipid injury to the GFB. Together, our results demonstrate that a multifunctional and modular microphysiological system can be deployed without the use of PDMS. Further, the bio-inert plastic used in our microfluidic device is amenable to various established, high-throughput manufacturing techniques, such as injection molding, to meet the increasing demand for organ-on-chip technology.

## Data Availability Statement

The data supporting the findings of this manuscript are available from the corresponding authors upon reasonable request.

## Author Contributions

MI designed the experiments and wrote the manuscript with the help of AF and AA. MI and JH designed and fabricated the MPS. MI, QA, and LW performed the cell culture. MI and AH performed the membrane strain experiments. AM, AS, and AF provided expertise, cells, and reagents for glomerular filtration experiments. All authors reviewed, edited, and approved the final version of the manuscript.

## Conflict of Interest

MI and AA are co-founders of Bio-Vitro, which is in the process of licensing the underlying IP for the microfluidic chip described. AF is an investor on pending or issued patents (US 10,183,038 and US 10,052,345) aimed at diagnosing or treating proteinuric kidney diseases. She stands to gain royalties from the future commercialization of these patents. She is Chief Scientific Officer of L&F Health LLC and is a consultant for Variant Pharmaceuticals. Variant Pharmaceuticals has licensed worldwide rights from L&F Research to develop and commercialize hydroxypropyl-beta-cyclodextrin for the treatment of kidney disease. The remaining authors declare that the research was conducted in the absence of any commercial or financial relationships that could be construed as a potential conflict of interest.
